# Benefits of Incretin Therapy on Ovarian Function: A Scientific Literature Review

**DOI:** 10.3390/ijms27114752

**Published:** 2026-05-25

**Authors:** Sandro La Vignera, Rosita A. Condorelli

**Affiliations:** Department of Clinical and Experimental Medicine, University of Catania, 95124 Catania, Italy; rosita.condorelli@unict.it

**Keywords:** GLP-1 receptor agonists, incretin therapy, polycystic ovary syndrome, PCOS, ovarian function, fertility, reproductive health, liraglutide, semaglutide, tirzepatide

## Abstract

Incretin-based therapies, particularly glucagon-like peptide-1 receptor agonists (GLP-1 RAs), have emerged as potentially promising therapeutic agents for improving ovarian function, especially in women with polycystic ovary syndrome (PCOS) and obesity-related reproductive dysfunction. This comprehensive review synthesizes evidence from 30 highly relevant studies examining the mechanisms of action, clinical outcomes, and safety profile of incretin therapies on ovarian function. The evidence suggests that GLP-1 RAs may exert beneficial effects through multiple molecular pathways, including FOXO1 signaling, modulation of steroidogenesis, and enhancement of insulin sensitivity, although most mechanistic data derive from animal models and in vitro studies without validation in human ovarian tissue. Clinical outcomes from randomized controlled trials and meta-analyses show improvements in menstrual regularity, hormonal profiles, and spontaneous conception rates, though evidence certainty is limited by small sample sizes, short duration, high heterogeneity, and restriction to overweight/obese populations. While preliminary safety data regarding inadvertent early pregnancy exposure are reassuring, animal studies suggest potential dose-dependent risks that warrant careful consideration. Importantly, GLP-1 RAs are not currently approved or guideline-recommended for fertility restoration, and substantial uncertainty remains regarding long-term reproductive safety, optimal patient selection, and clinical guidelines. This review provides a balanced synthesis of current evidence and identifies critical gaps requiring further investigation before routine clinical use can be recommended.

## 1. Introduction

Polycystic ovary syndrome (PCOS) affects 5–20% of women of reproductive age and represents the most common endocrine disorder associated with anovulation and infertility [1]. The syndrome is characterized by hyperandrogenism, irregular menstrual cycles, polycystic ovarian morphology, and frequently coexists with obesity, insulin resistance, and metabolic dysfunction [2]. Traditional management strategies have focused on lifestyle modifications, metformin, and ovulation induction agents, yet many women continue to experience suboptimal reproductive outcomes [3].

Incretin-based therapies, originally developed for type 2 diabetes management, have garnered increasing attention for their potential benefits in ovarian function and reproductive health [4,5]. These agents include glucagon-like peptide-1 receptor agonists (GLP-1 RAs) such as liraglutide, semaglutide, exenatide, and dulaglutide, as well as dual glucose-dependent insulinotropic polypeptide (GIP)/GLP-1 receptor agonists such as tirzepatide [6,7]. Beyond their well-established effects on glycemic control and weight reduction, emerging evidence suggests that GLP-1 RAs may directly influence ovarian physiology through receptor-mediated mechanisms [1].

It is important to note that while mechanistic insights from preclinical models are substantial, the clinical evidence base includes a limited number of high-quality randomized controlled trials (RCTs), with much of the literature comprising narrative reviews, observational studies, and hypothesis-generating investigations. This review explicitly distinguishes between established clinical evidence and preliminary or mechanistic data throughout. Furthermore, it is essential to emphasize that GLP-1 RAs are not currently approved or guideline-recommended for fertility restoration or PCOS management. The evidence reviewed herein is preliminary and hypothesis-generating, and substantial uncertainty remains regarding long-term reproductive outcomes, pregnancy safety, and optimal patient selection.

This comprehensive review synthesizes current evidence on the benefits of incretin therapy for ovarian function, with particular emphasis on molecular mechanisms, clinical reproductive outcomes, and safety considerations. We examine data from systematic reviews, randomized controlled trials, prospective studies, and mechanistic investigations to provide a thorough understanding of this evolving therapeutic approach while highlighting areas of uncertainty and the need for rigorous clinical trials.

## 2. Background and Theoretical Foundations

The rationale for using incretin-based therapies in reproductive disorders is supported by multiple converging lines of evidence. First, GLP-1 receptors have been identified in human ovarian tissue, including granulosa cells, theca cells, and oocytes, suggesting direct ovarian effects independent of systemic metabolic improvements [1]. Second, the strong association between obesity, insulin resistance, and reproductive dysfunction in PCOS provides a mechanistic basis for interventions that address these metabolic abnormalities [8,9].

While PCOS exhibits substantial phenotypic heterogeneity and affects both lean and obese women, obesity is a common comorbidity that exacerbates metabolic and reproductive dysfunction in affected individuals [10,11]. In obese women with PCOS, adipose tissue dysfunction contributes to chronic inflammation, hyperinsulinemia, and androgen excess, creating a bidirectional relationship between metabolic and reproductive pathology [9]. Importantly, approximately 20–30% of women with PCOS are of normal weight, and the pathophysiology in lean PCOS may differ substantially from that in obese phenotypes [12]. While obesity significantly exacerbates the metabolic and reproductive features of PCOS, it is important to note that the syndrome is not synonymous with a complication of obesity. Rather, obesity and PCOS share bidirectional pathophysiological relationships.

The incretin system plays a crucial role in glucose homeostasis through enhancement of glucose-dependent insulin secretion, suppression of glucagon release, slowing of gastric emptying, and promotion of satiety [4]. GLP-1 RAs mimic the actions of endogenous GLP-1, which is rapidly degraded by dipeptidyl peptidase-4 (DPP-4). These agents have demonstrated robust efficacy in improving glycemic control and promoting weight loss in individuals with type 2 diabetes and obesity [6].

The discovery of GLP-1 receptors in reproductive tissues has prompted investigation into potential direct effects on ovarian function beyond systemic metabolic improvements [1]. Proposed mechanisms include modulation of steroidogenesis, enhancement of insulin sensitivity in ovarian cells, reduction in oxidative stress and inflammation, and regulation of follicular development and ovulation [13]. It is critical to emphasize that most molecular pathway evidence derives from animal models (primarily rodent PCOS models) and in vitro cellular experiments. Validation of these pathways in human ovarian tissue or clinical samples remains limited, and extrapolation from preclinical systems to human reproductive physiology must be undertaken with caution.

### 2.1. Search Strategy and Methodology

#### 2.1.1. Literature Search Strategy

A comprehensive literature search was conducted across four major databases: PubMed/MEDLINE, Scopus, Web of Science, and Google Scholar. The search was performed in March 2026 and covered publications from January 2015 to March 2026. The following search terms were used in combination: (“GLP-1 receptor agonist” OR “glucagon-like peptide-1” OR “liraglutide” OR “semaglutide” OR “exenatide” OR “dulaglutide” OR “tirzepatide” OR “incretin therapy” OR “incretin”) AND (“polycystic ovary syndrome” OR “PCOS” OR “ovarian function” OR “reproductive health” OR “fertility” OR “ovulation” OR “menstrual” OR “pregnancy” OR “conception”).

#### 2.1.2. Inclusion and Exclusion Criteria

Studies were included if they: (1) investigated GLP-1 receptor agonists, dual GIP/GLP-1 agonists, or other incretin-based therapies; (2) reported outcomes related to ovarian function, reproductive hormones, menstrual cyclicity, ovulation, or fertility; (3) included human participants (clinical trials, observational studies, systematic reviews, meta-analyses) or mechanistic studies (animal models, in vitro experiments); and (4) were published in English in peer-reviewed journals between 2015 and 2025. Exclusion criteria included: (1) studies focusing solely on metabolic outcomes without reproductive endpoints; (2) conference abstracts without full-text availability; and (3) duplicate publications.

#### 2.1.3. Study Selection and Data Extraction

Two authors independently screened titles and abstracts, followed by full-text review of potentially eligible studies. Disagreements were resolved through discussion. Data extracted included study design, population characteristics, intervention details, outcome measures, and key findings. A total of 30 highly relevant studies were selected for detailed synthesis, including 5 systematic reviews/meta-analyses [13,14,15,16], 8 randomized controlled trials [17,18,19,20,21], 7 prospective cohort studies, 6 mechanistic animal studies, and 4 in vitro investigations.

#### 2.1.4. Quality Assessment

The quality of randomized controlled trials was assessed using the Cochrane Risk of Bias tool (RoB 2). Systematic reviews were evaluated using AMSTAR 2 (A MeaSurement Tool to Assess systematic Reviews). Observational studies were appraised using the Newcastle-Ottawa Scale. Mechanistic studies were evaluated for methodological rigor, reproducibility, and translational relevance. While formal risk-of-bias assessment using standardized tools was not conducted for all studies given the narrative nature of this review, we critically appraised included studies based on study design, sample size, randomization and blinding (for RCTs), completeness of outcome data, selective outcome reporting, confounding control (for observational studies), and translational applicability (for preclinical studies). Study limitations are discussed in the context of each finding throughout the manuscript.

As a narrative review without formal systematic methodology, we did not conduct quantitative assessment of publication bias (e.g., funnel plots, Egger’s test). However, we acknowledge that publication bias is a potential threat to the validity of our conclusions, particularly given the predominance of small, short-term studies and the emerging, highly publicized nature of GLP-1 RAs for metabolic and reproductive health. Readers should interpret the evidence with this limitation in mind.

## 3. Molecular Mechanisms: Evidence from Preclinical Models

The molecular pathways through which GLP-1 receptor agonists may influence ovarian function have been investigated primarily in animal models and in vitro systems. While these mechanistic studies provide valuable insights into potential therapeutic targets, it is critical to note that the pathways described below have not yet been validated in human ovarian tissue or clinical samples. Translational validation remains a key priority for future research.

### 3.1. FOXO1 Signaling Pathway

Forkhead box O1 (FOXO1) is a transcription factor that plays a critical role in regulating granulosa cell proliferation, apoptosis, and steroidogenesis [1]. Evidence from dehydroepiandrosterone (DHEA)-induced mouse models [animal study] and in vitro granulosa cell experiments [in vitro] demonstrates that GLP-1 receptor activation modulates FOXO1 phosphorylation, promoting granulosa cell proliferation and reducing apoptosis [1]. GLP-1 RAs appear to enhance phosphorylation of FOXO1, leading to its cytoplasmic retention and reduced transcriptional activity, which may improve follicular development and reduce follicular atresia.

Validation Status: FOXO1 pathway modulation by GLP-1 receptor agonists has been demonstrated in DHEA-induced mouse PCOS models and in vitro experiments using mouse and human granulosa cells (DOI: 10.1155/2020/1484321) [1]. However, validation in human ovarian tissue samples from women with PCOS treated with GLP-1 RAs has not been reported. The translational relevance of these findings to clinical ovarian function remains to be established.

### 3.2. PI3K/Akt/mTOR Pathway

The phosphoinositide 3-kinase (PI3K)/protein kinase B (Akt)/mammalian target of rapamycin (mTOR) signaling pathway regulates cellular metabolism, growth, and survival [13]. Semaglutide reduced ovarian oxidative stress and autophagy via PI3K/Akt/mTOR signaling in mouse PCOS models [animal study], suggesting that GLP-1 RAs may protect ovarian cells from oxidative damage and improve cellular homeostasis [13]. Activation of this pathway may enhance insulin signaling in ovarian cells, improve glucose uptake, and reduce cellular stress responses.

Validation Status: Evidence for PI3K/Akt/mTOR pathway involvement comes exclusively from mouse PCOS models treated with semaglutide (DOI: 10.2147/DDDT.S522730) [13]. No studies have validated this pathway in human ovarian cells or clinical samples. Given known species differences in ovarian physiology and insulin signaling, human validation is essential before clinical extrapolation.

### 3.3. AMPK/SIRT1/NF-κB Axis

AMP-activated protein kinase (AMPK) is a master regulator of cellular energy homeostasis, while sirtuin 1 (SIRT1) is a NAD+-dependent deacetylase involved in metabolic regulation and inflammation [13]. Nuclear factor-kappa B (NF-κB) is a key transcription factor mediating inflammatory responses. Semaglutide ameliorated ovarian inflammation through the AMPK/SIRT1/NF-κB axis in PCOS mice [animal study], reducing pro-inflammatory cytokine expression and improving ovarian histology [13]. This anti-inflammatory mechanism may contribute to improved follicular development and reduced ovarian dysfunction in PCOS.

Validation Status: The anti-inflammatory effects of semaglutide via AMPK/SIRT1/NF-κB signaling have been demonstrated in PCOS mouse models (DOI: 10.2147/DDDT.S484531) [13]. Human ovarian tissue validation is lacking. While systemic anti-inflammatory effects of GLP-1 RAs are well-documented in human metabolic studies, direct ovarian anti-inflammatory mechanisms remain unconfirmed in clinical samples.

### 3.4. BMP4-Smad Signaling

Bone morphogenetic protein 4 (BMP4) and its downstream Smad signaling pathway regulate ovarian steroidogenesis and follicular development [13]. GLP-1 RAs activated BMP4-Smad signaling and reduced ovarian androgen synthesis in mouse PCOS experiments [animal study] (DOI: 10.1093/biolre/ioag065) [13]. This mechanism may contribute to the reduction in hyperandrogenism observed in clinical studies of GLP-1 RAs in women with PCOS.

Validation Status: BMP4-Smad pathway activation by GLP-1 RAs has been shown in mouse PCOS experiments (DOI: 10.1093/biolre/ioag065) [13]. Human ovarian cell validation has not been published. BMP signaling is known to regulate human ovarian steroidogenesis, but whether GLP-1 RAs modulate this pathway in human granulosa or theca cells is unknown.

### 3.5. GPX4 and Ferroptosis

Glutathione peroxidase 4 (GPX4) is a critical antioxidant enzyme that protects cells from ferroptosis, an iron-dependent form of regulated cell death characterized by lipid peroxidation [13]. Semaglutide reduced ovarian ferroptosis and GPX4 promoter hypermethylation in a mouse model [animal study] (DOI: 10.1007/s10735-026-10764-9) [13]. By preventing ferroptosis, GLP-1 RAs may protect ovarian cells from oxidative damage and improve follicular health.

Validation Status: Semaglutide reduced ovarian ferroptosis and GPX4 promoter hypermethylation in a mouse model (DOI: 10.1007/s10735-026-10764-9) [13]. The authors of that study explicitly noted that translational validation in human ovarian samples is needed. No human ovarian tissue studies have examined GLP-1 RA effects on ferroptosis or GPX4 expression.

### 3.6. Modulation of Steroidogenesis and Androgen Synthesis

GLP-1 receptors are expressed in human ovarian granulosa and theca cells, suggesting potential direct effects on steroidogenesis [1]. Preclinical studies [animal studies] suggest that GLP-1 RAs may reduce androgen synthesis by modulating steroidogenic enzyme expression, including cytochrome P450 17A1 (CYP17A1) and 3β-hydroxysteroid dehydrogenase (3β-HSD) [13]. This mechanism may contribute to the reduction in testosterone and improvement in androgen-related symptoms observed in clinical studies.

Validation Status: While GLP-1 receptor expression has been confirmed in human ovarian tissue [1], direct mechanistic studies examining GLP-1 RA effects on steroidogenic enzyme expression or androgen synthesis in human granulosa or theca cells are lacking. Most mechanistic data derive from animal models [animal studies].

#### Summary of Molecular Pathway Validation

Table following (Table 1) summarizes the main mechanisms through which incretin-based therapies influence ovarian function.

## 4. Clinical Outcomes in Human Studies

### 4.1. Evidence from Systematic Reviews and Meta-Analyses

The highest level of synthesized evidence comes from systematic reviews and meta-analyses examining GLP-1 RA effects on reproductive outcomes in women with PCOS.

#### 4.1.1. Menstrual Cyclicity and Ovulation

A comprehensive meta-analysis by Zhou et al. (2023) [meta-analysis] [16] pooled data from 12 studies and found that GLP-1 RAs significantly improved menstrual cyclicity in women with PCOS compared to control interventions. However, this analysis revealed very high heterogeneity (I^2^ = 95.6%), indicating substantial inconsistency across studies and necessitating cautious interpretation. The authors noted that approximately half of included studies had “some concerns” in randomization or other bias domains. Despite these limitations, the overall trend suggested benefit in restoring regular menstrual cycles.

#### 4.1.2. Pregnancy and Fertility Outcomes

The same meta-analysis [meta-analysis] [16] reported improvements in spontaneous pregnancy rates among women with PCOS treated with GLP-1 RAs compared to controls. However, pregnancy was typically a secondary outcome in most trials, and studies were not adequately powered to detect differences in pregnancy rates as a primary endpoint. The evidence certainty for pregnancy outcomes was graded as low to very low due to imprecision, indirectness, and risk of bias.

A systematic review by Pugliese et al. (2023) [systematic review] [22] examined whether the reproductive benefits of liraglutide in PCOS are mediated solely by weight loss or include weight-independent mechanisms. The review concluded that while weight loss is a principal mediator of many observed endocrine improvements, some metabolic and hormonal benefits may occur independent of weight reduction, potentially through direct ovarian effects or improved insulin sensitivity.

#### 4.1.3. Hormonal Profiles

Multiple systematic reviews [systematic reviews] [14,15] have documented improvements in hormonal profiles with GLP-1 RA therapy in women with PCOS, including: reduction in total testosterone and free androgen index; improvement in sex hormone-binding globulin (SHBG) levels; reduction in luteinizing hormone (LH) and LH/follicle-stimulating hormone (FSH) ratio; and improvement in anti-Müllerian hormone (AMH) levels in some studies.

However, these hormonal improvements are closely correlated with weight loss and improved insulin sensitivity, making it difficult to disentangle direct ovarian effects from systemic metabolic improvements.

### 4.2. Evidence from Randomized Controlled Trials

Individual RCTs provide direct experimental evidence for GLP-1 RA effects on reproductive outcomes.

#### 4.2.1. Liraglutide in PCOS

The RCT by Nylander et al. (2017) [RCT] [20] demonstrated improvements in menstrual regularity and ovulation frequency with liraglutide (1.8 mg daily) versus placebo in 72 women with PCOS over 26 weeks. Strengths of this trial include randomized, placebo-controlled design and objective outcome measures (menstrual diaries, ovulation confirmed by progesterone levels). However, limitations include small sample size (*n* = 72), short duration (26 weeks), and enrollment restricted to overweight/obese women (BMI ≥ 25 kg/m^2^), limiting generalizability to lean PCOS phenotypes.

#### 4.2.2. Semaglutide Combined with Metformin

A recent RCT (2025) [RCT] [21] examined combined semaglutide and metformin therapy versus metformin alone in 120 overweight/obese women with PCOS over 24 weeks. The combination therapy group showed significant improvements in body weight, metabolic parameters (fasting glucose, insulin resistance), hormonal profiles (testosterone, LH/FSH ratio), and spontaneous pregnancy rates compared to metformin alone. While promising, this trial was open-label (not blinded), which may introduce performance and detection bias, and pregnancy was a secondary outcome not powered as a primary endpoint.

#### 4.2.3. Liraglutide in Assisted Reproduction

Several RCTs [RCTs] [17,18,19] have examined liraglutide as an adjunct to in vitro fertilization (IVF) or intracytoplasmic sperm injection (ICSI) in women with PCOS. These studies reported improvements in oocyte quality, embryo quality, and clinical pregnancy rates in liraglutide-treated groups compared to controls. However, sample sizes were small (typically 40–80 participants per study), and results were inconsistent across trials.

#### 4.2.4. Critical Appraisal of RCT Evidence

While these clinical outcomes are encouraging, several important caveats must be emphasized:

1. Short duration: Most trials are short-term (3–6 months), and long-term reproductive outcomes and pregnancy safety data are limited.
2. Small sample sizes: Many trials enroll fewer than 100 participants, limiting statistical power and precision of effect estimates.
3. Methodological concerns: Approximately half of included RCTs have “some concerns” or “high risk” in one or more bias domains, particularly randomization, allocation concealment, blinding, and selective outcome reporting.
4. Population restriction: The majority of evidence comes from overweight and obese PCOS populations, and benefits in lean PCOS or other reproductive disorders remain uncertain.
5. Guideline status: Current clinical guidelines from the American Society for Reproductive Medicine (ASRM), the European Society of Human Reproduction and Embryology (ESHRE), and the International PCOS Network do not endorse GLP-1 RAs for fertility restoration, and their use in this context remains off-label and investigational.

Clinicians considering GLP-1 RA therapy for reproductive indications should engage in shared decision-making with patients, emphasizing the preliminary nature of the evidence and the importance of contraception counseling given limited pregnancy safety data.

### 4.3. Evidence from Prospective Cohort Studies

Observational studies complement RCT data by examining real-world effectiveness and longer-term outcomes. Several prospective cohort studies [cohort studies] have reported improvements in menstrual regularity, spontaneous ovulation, and pregnancy rates among women with PCOS treated with GLP-1 RAs in clinical practice settings [23,24]. However, these studies are subject to confounding by indication, as women receiving GLP-1 RAs often differ systematically from controls in baseline BMI, metabolic profile, and motivation for lifestyle change. Adjustment for confounders is often incomplete, limiting causal inference.

### 4.4. Case Reports and Case Series

Isolated case reports [case reports], while hypothesis-generating, represent the lowest level of clinical evidence. Several case reports have described unintended pregnancies during GLP-1 RA therapy in women with PCOS who had previously experienced infertility [25,26]. This phenomenon—potentially related to improved ovulatory function and reduced efficacy of oral contraceptives due to delayed gastric emptying—underscores the critical importance of contraception counseling for women of reproductive age receiving GLP-1 RAs.

## 5. Safety Considerations and Pregnancy Exposure

### 5.1. Pregnancy Safety Data

Current pregnancy safety data for GLP-1 RAs are limited and derive primarily from inadvertent early pregnancy exposures in diabetes and obesity trials, manufacturer registries, and case reports [case reports, registry data]. While preliminary human data have not identified major teratogenic signals, these data are insufficient to establish safety, and animal reproductive toxicology studies have demonstrated dose-dependent embryo-fetal toxicity.

#### 5.1.1. Animal Reproductive Toxicology

Animal studies [animal studies] in rats and rabbits have demonstrated dose-dependent adverse effects on embryo-fetal development with GLP-1 RAs, including: increased early pregnancy loss and resorptions; skeletal malformations and delayed ossification; reduced fetal body weight and growth restriction; and increased incidence of visceral and skeletal variations.

These effects typically occur at exposures comparable to or slightly higher than human therapeutic exposures, raising concerns about potential risks during human pregnancy.

#### 5.1.2. Human Pregnancy Exposure Data

Preliminary human data from inadvertent early pregnancy exposures [case reports, registry data] [26] have not identified a clear pattern of major congenital malformations. However, these data are limited by: small numbers of exposed pregnancies; lack of systematic follow-up and outcome ascertainment; potential underreporting of adverse outcomes; confounding by maternal obesity, diabetes, and PCOS.

A recent review [narrative review] [26] of pregnancy exposures to GLP-1 RAs (primarily semaglutide and liraglutide) found no consistent teratogenic signal but emphasized the preliminary nature of the data and the need for larger, prospective pregnancy registries.

#### 5.1.3. Current Prescribing Recommendations

Current prescribing information for all approved GLP-1 RAs recommends: discontinuation at least 2 months before planned conception for weekly formulations (semaglutide, dulaglutide); discontinuation at least 1 month before planned conception for daily formulations (liraglutide); use of effective contraception during treatment; and immediate discontinuation if pregnancy is detected.

### 5.2. Contraception Counseling

Women of reproductive age receiving GLP-1 RAs for metabolic indications should be counseled regarding:

1. Potential for restored ovulation: GLP-1 RAs may restore ovulatory function in women with PCOS who were previously anovulatory, increasing pregnancy risk.
2. Reduced oral contraceptive efficacy: Delayed gastric emptying caused by GLP-1 RAs may reduce absorption and efficacy of oral contraceptives. Alternative contraceptive methods (intrauterine devices, implants, barrier methods) should be considered.
3. Preconception planning: Women planning pregnancy should discontinue GLP-1 RAs 1–2 months before conception attempts and transition to pregnancy-safe alternatives for glycemic control if needed (e.g., insulin).
4. Immediate discontinuation if pregnancy occurs: If pregnancy is detected during GLP-1 RA therapy, the medication should be discontinued immediately, and the patient should be referred for prenatal care and counseling.

### 5.3. Other Safety Considerations

GLP-1 RAs are generally well-tolerated, with the most common adverse effects being gastrointestinal (nausea, vomiting, diarrhea), which typically diminish over time with dose titration. Rare but serious adverse effects include pancreatitis, gallbladder disease, and thyroid C-cell tumors (observed in rodent studies but not confirmed in humans). Women with personal or family history of medullary thyroid carcinoma or multiple endocrine neoplasia syndrome type 2 should not receive GLP-1 RAs.

## 6. Tirzepatide and Emerging Multi-Receptor Agonists

### 6.1. Rationale for Dual GIP/GLP-1 Receptor Agonism

Tirzepatide is a novel dual glucose-dependent insulinotropic polypeptide (GIP) and GLP-1 receptor agonist that has demonstrated superior weight loss and glycemic control compared to selective GLP-1 receptor agonists in type 2 diabetes trials [27]. The dual mechanism of action—combining GLP-1-mediated insulin secretion, appetite suppression, and gastric emptying delay with GIP-mediated enhancement of insulin sensitivity and potential direct metabolic effects—provides a strong theoretical rationale for application in obesity-related PCOS [28].

### 6.2. Preclinical Evidence in PCOS Models

A recent study examined tirzepatide in a testosterone-propionate-induced rat PCOS model [animal study] and demonstrated dose-dependent improvements in reproductive hormones (reduced LH, testosterone, and LH/FSH ratio), ovarian histology (increased corpus luteum number, reduced cystic follicles), metabolic indices (improved insulin sensitivity, reduced body weight), and ovarian inflammation markers (DOI: 10.1007/s00210-026-05346-1) [29]. These effects were comparable to metformin in that preclinical model. However, it is critical to emphasize that these findings are derived from a single animal study, and no controlled clinical trials of tirzepatide in women with PCOS have been published.

### 6.3. Human Evidence: Case Reports and Narrative Reviews

Human evidence for tirzepatide in PCOS is currently limited to isolated case reports and narrative reviews. One case report [case report] described spontaneous conception after sequential treatment with semaglutide and tirzepatide in an obese woman with PCOS and type 2 diabetes. While this observation is hypothesis-generating, it represents anecdotal evidence and cannot establish causality or generalizability.

Narrative reviews [narrative reviews] have highlighted tirzepatide’s mechanism of action and substantial weight loss efficacy as making it a plausible therapeutic option for obese PCOS patients (DOI: 10.3390/jcm12144575) [30]. However, these reviews consistently emphasize the absence of controlled PCOS trials and the urgent need for safety and fertility-specific studies before routine clinical use, especially in normal-weight patients [30].

### 6.4. Evidence Gaps and Research Priorities

The current evidence base for tirzepatide in PCOS is characterized by:

1. Absence of controlled clinical trials: No randomized controlled trials, prospective cohort studies, or systematic observational studies have examined tirzepatide’s effects on reproductive outcomes, menstrual cyclicity, ovulation rates, or fertility in women with PCOS.
2. Unknown safety profile in pregnancy: Given the mechanism of action and the potential for unintended pregnancy in women with restored ovulation, safety data regarding early pregnancy exposure are critically needed.
3. Uncertain applicability to lean PCOS: The substantial weight loss associated with tirzepatide raises questions about its appropriateness and safety in normal-weight women with PCOS.
4. Lack of comparative effectiveness data: No head-to-head trials have compared tirzepatide to established PCOS treatments (metformin, letrozole, lifestyle modification) or to selective GLP-1 receptor agonists.

### 6.5. Future Multi-Receptor Agonists

Beyond tirzepatide, triple agonists targeting GLP-1, GIP, and glucagon receptors (e.g., retatrutide) are in clinical development for obesity and metabolic disease [31]. While these agents show promise for metabolic outcomes, their potential role in reproductive health is entirely speculative at present. The field would benefit from mechanistic studies examining GIP receptor expression and function in human ovarian tissue, as well as preclinical studies of multi-receptor agonists in validated PCOS models.

### 6.6. Clinical Implications and Recommendations

Based on the current evidence, tirzepatide cannot be recommended for routine treatment of PCOS outside of clinical trial settings. While the preclinical data are encouraging and the mechanism of action is plausible, the absence of controlled human trials, safety data, and comparative effectiveness evidence precludes evidence-based clinical use. Clinicians considering off-label use should:

1. Limit use to obese women with PCOS and comorbid type 2 diabetes (where tirzepatide is indicated for glycemic control).
2. Provide comprehensive contraceptive counseling given the potential for restored ovulation.
3. Closely monitor reproductive and metabolic outcomes.
4. Participate in registry studies or prospective data collection to build the evidence base.

Dedicated randomized controlled trials of tirzepatide in PCOS, with reproductive endpoints (menstrual cyclicity, ovulation rate, pregnancy rate) and adequate safety follow-up, are urgently needed.

## 7. Comparative Effectiveness and Clinical Context

### 7.1. Comparison with Metformin

Metformin remains the most widely studied and recommended pharmacological intervention for metabolic dysfunction in PCOS [3]. Several studies [RCTs, meta-analyses] have compared GLP-1 RAs to metformin or examined combination therapy.

Metabolic Outcomes: GLP-1 RAs generally produce greater weight loss than metformin, with liraglutide and semaglutide achieving 5–15% body weight reduction compared to 2–5% with metformin [14,22]. Improvements in insulin sensitivity are comparable between the two classes, though mechanisms differ (peripheral insulin sensitization with metformin vs. weight loss-mediated improvement with GLP-1 RAs).

Reproductive Outcomes: Limited head-to-head data suggest similar improvements in menstrual regularity and ovulation rates with GLP-1 RAs and metformin, though GLP-1 RAs may produce more rapid improvements due to greater weight loss [22]. Combination therapy (GLP-1 RA plus metformin) may offer additive benefits [RCT] [21,32].

Safety and Tolerability: Both classes are generally well-tolerated, with gastrointestinal side effects being most common. Metformin has a longer safety track record and established safety in pregnancy (FDA category B), whereas GLP-1 RAs require preconception discontinuation due to limited pregnancy safety data.

Cost and Accessibility: Metformin is generic and inexpensive, whereas GLP-1 RAs are costly branded medications with variable insurance coverage. Cost-effectiveness analyses are needed to inform clinical decision-making.

### 7.2. Comparison with Ovulation Induction Agents

Letrozole and clomiphene citrate are first-line ovulation induction agents for women with PCOS-related infertility [3]. GLP-1 RAs are not intended to replace these agents but may serve as adjunctive therapy to optimize metabolic health before or during ovulation induction.

Sequential Approach: A rational approach may involve initial metabolic optimization with lifestyle modification and/or GLP-1 RA therapy (with appropriate contraception), followed by discontinuation of GLP-1 RA and initiation of ovulation induction agents once metabolic goals are achieved. This approach requires validation in prospective trials.

### 7.3. Role in Assisted Reproductive Technology

Several studies [RCTs] [17,18,19] have examined GLP-1 RAs as adjuncts to IVF/ICSI in women with PCOS, with mixed results. Potential benefits include improved oocyte and embryo quality, though effects on live birth rates remain uncertain. Current evidence is insufficient to recommend routine use of GLP-1 RAs in assisted reproduction protocols.

## 8. Discussion

### 8.1. Synthesis of Evidence

This comprehensive review synthesizes evidence from 30 studies examining incretin-based therapies for ovarian function, with particular focus on GLP-1 receptor agonists in women with PCOS. The evidence suggests that GLP-1 RAs may improve reproductive outcomes through multiple mechanisms, including systemic metabolic improvements (weight loss, enhanced insulin sensitivity, reduced inflammation) and potential direct ovarian effects mediated by GLP-1 receptors expressed in ovarian tissue.

Clinical evidence from systematic reviews, meta-analyses, and RCTs indicates improvements in menstrual regularity, hormonal profiles, and spontaneous conception rates with GLP-1 RA therapy. However, the evidence base is characterized by significant limitations, including small sample sizes, short duration, high heterogeneity, methodological concerns, and restriction to overweight/obese populations.

Mechanistic studies in animal models and in vitro systems have identified multiple molecular pathways (FOXO1, PI3K/Akt/mTOR, AMPK/SIRT1/NF-κB, BMP4-Smad, GPX4/ferroptosis) through which GLP-1 RAs may influence ovarian function. However, none of these pathways have been validated in human ovarian tissue or clinical samples, and translational applicability remains uncertain.

### 8.2. Evidence Quality and Translational Gaps

A critical appraisal of the evidence base reveals important limitations:

1. Mechanistic pathway data are derived almost entirely from animal models and in vitro experiments, with no published validation in human ovarian tissue samples. Rodent PCOS models do not fully recapitulate the complex endocrine, metabolic, and genetic heterogeneity of human PCOS, and GLP-1 RA doses used in animal studies often exceed weight-adjusted human equivalent doses.
2. Clinical trial evidence is characterized by high heterogeneity (I^2^ ≈ 95.6% for menstrual cyclicity outcomes in meta-analysis) [16], small sample sizes, and short follow-up durations. Approximately half of included RCTs exhibited methodological concerns in randomization, allocation concealment, blinding, or outcome reporting domains.
3. Most RCTs enrolled overweight or obese women, limiting generalizability to lean PCOS phenotypes. Approximately 20–30% of women with PCOS are of normal weight, and the pathophysiology and treatment response in lean PCOS may differ substantially from obese phenotypes.
4. Evidence certainty for several comparisons has been graded as very low in recent systematic reviews, reflecting concerns about study quality, imprecision, indirectness, and inconsistency.

These limitations underscore the need for larger, longer, and more rigorously designed trials with reproductive endpoints and safety follow-up.

### 8.3. Applicability to Lean PCOS Phenotypes

A critical limitation of the current evidence base is the predominant focus on overweight and obese PCOS populations. Approximately 20–30% of women with PCOS are of normal weight, and the pathophysiology, metabolic profile, and treatment response in lean PCOS may differ substantially from obese phenotypes [12]. Most clinical trials of GLP-1 receptor agonists have enrolled overweight or obese participants, and weight loss is a principal mediator of many observed endocrine improvements [14,15]. There is currently insufficient evidence to recommend GLP-1 RAs for reproductive or metabolic treatment in lean PCOS, and dedicated trials in lean/normal-weight cohorts are urgently needed. Clinicians should exercise caution in extrapolating benefits observed in obese cohorts to lean patients, and alternative therapeutic strategies (e.g., inositol supplementation, letrozole for ovulation induction) may be more appropriate first-line options in this population.

### 8.4. Publication Bias and Reporting Limitations

Publication bias represents a significant potential threat to the validity of the evidence base on GLP-1 RAs for reproductive outcomes. Several factors suggest that publication bias may be present in this literature:

1. Small, short-term studies: The field is characterized by predominantly small, short-term studies, which are particularly susceptible to publication bias, as small studies with null or negative results are less likely to be published than those with positive findings.
2. Industry sponsorship: Many studies are industry-sponsored or conducted by investigators with financial relationships with GLP-1 RA manufacturers, which may influence study design, outcome selection, and publication decisions.
3. Emerging and highly publicized field: The emerging and highly publicized nature of GLP-1 RAs for weight loss and metabolic health may create publication incentives favoring positive reproductive outcomes.
4. Lack of formal publication bias assessment: Systematic reviews in this field have not consistently performed or reported formal publication bias assessments (e.g., funnel plots, Egger’s test), limiting the ability to quantitatively evaluate this threat.

Importantly, available systematic reviews and meta-analyses have not consistently documented publication bias using formal statistical methods. For example, while Zhou et al. [16] conducted a comprehensive meta-analysis of GLP-1 RAs on pregnancy rate and menstrual cyclicity, formal funnel plot analysis or statistical tests for small-study effects were not reported. Similarly, other systematic reviews in this field have not routinely included publication bias assessment in their methodology. This represents a significant gap in the evidence synthesis literature.

The potential impact of publication bias on the conclusions of this review is difficult to quantify but could be substantial. If small negative or null studies remain unpublished, the apparent benefits of GLP-1 RAs on menstrual regularity, ovulation, and pregnancy rates may be overestimated. Conversely, safety signals or adverse reproductive outcomes may be underreported.

To mitigate publication bias in future evidence synthesis, we recommend: (1) prospective trial registration in public registries (e.g., ClinicalTrials.gov) with mandatory results reporting regardless of outcome; (2) routine inclusion of funnel plots and statistical tests for publication bias in systematic reviews and meta-analyses; (3) efforts to identify and include unpublished data, conference abstracts, and trial registry results; (4) encouragement of journals to publish well-designed studies with null or negative results; and (5) transparency regarding funding sources and conflicts of interest.

### 8.5. Clinical Implications and Guideline Status

From a clinical perspective, it is essential to emphasize that GLP-1 RAs are not currently approved or recommended by professional guidelines for fertility restoration or PCOS management. The American Society for Reproductive Medicine (ASRM), the European Society of Human Reproduction and Embryology (ESHRE), and the International PCOS Network do not include GLP-1 RAs in their evidence-based treatment algorithms for PCOS-related infertility. The evidence base, while promising, remains insufficient to support routine clinical use outside of research protocols.

Key uncertainties include: 1. Long-term reproductive outcomes and pregnancy safety. 2. Optimal patient selection criteria (BMI thresholds, PCOS phenotype, metabolic profile). 3. Optimal dosing, duration, and timing relative to conception attempts. 4. Comparative effectiveness versus established therapies (metformin, letrozole, lifestyle modification). 5. Cost-effectiveness.

Until these questions are addressed through adequately powered, long-term RCTs with reproductive endpoints and pregnancy registries, GLP-1 RA use for fertility restoration should be considered investigational and limited to research settings or highly selected clinical scenarios with informed consent and close monitoring.

## 9. Critical Appraisal and Publication Bias

### 9.1. Study Quality and Methodological Limitations

Critical appraisal of included studies reveals several methodological concerns that limit confidence in the evidence base:

Randomized Controlled Trials: Small sample sizes: Many trials enroll fewer than 100 participants, limiting statistical power and precision of effect estimates. Short duration: Most trials are 3–6 months in duration, providing limited information on long-term reproductive outcomes, pregnancy rates, or live birth rates. Lack of blinding: Several trials used open-label designs, which may introduce performance and detection bias. Methodological concerns: Approximately half of included RCTs have “some concerns” or “high risk” in one or more bias domains (randomization, allocation concealment, blinding, incomplete outcome data, selective reporting). Population restriction: Most trials enrolled overweight or obese women, limiting generalizability to lean PCOS phenotypes.

Systematic Reviews and Meta-Analyses: High heterogeneity: Meta-analytic estimates for some reproductive outcomes exhibit very high heterogeneity (e.g., menstrual cyclicity I^2^ = 95.6%), indicating substantial inconsistency across studies and necessitating cautious interpretation. Very low evidence certainty: Evidence certainty for some comparisons has been graded as very low, reflecting concerns about study quality, imprecision, indirectness, and inconsistency. Lack of publication bias assessment: Most systematic reviews have not performed or reported formal publication bias assessments (funnel plots, Egger’s test).

Observational Studies: Confounding by indication: Women receiving GLP-1 RAs often differ systematically from controls in baseline BMI, metabolic profile, and motivation for lifestyle change. Incomplete adjustment: Adjustment for confounders is often incomplete, limiting causal inference. Selection bias: Observational studies may be subject to selection bias, as women who receive GLP-1 RAs may differ from those who do not in unmeasured ways.

Preclinical Mechanistic Studies: Species differences: Rodent PCOS models do not fully recapitulate the complex endocrine, metabolic, and genetic heterogeneity of human PCOS. Artificial induction models: DHEA- or testosterone-induced PCOS models may not reflect the natural pathophysiology of human PCOS. Supraphysiologic doses: GLP-1 RA doses used in animal studies often exceed weight-adjusted human equivalent doses. Lack of human validation: None of the molecular pathways identified in animal models have been validated in human ovarian tissue or clinical samples.

### 9.2. Predominance of Obese/Overweight Populations

A critical limitation of the current evidence base is the predominant focus on overweight and obese PCOS populations. Most RCTs have enrolled women with BMI ≥ 25 kg/m^2^, and many have required BMI ≥ 30 kg/m^2^ for inclusion. This population restriction limits generalizability to lean PCOS phenotypes, which represent approximately 20–30% of women with PCOS. The pathophysiology, metabolic profile, and treatment response in lean PCOS may differ substantially from obese phenotypes, and weight loss-mediated mechanisms may be less relevant in normal-weight women. Dedicated trials in lean/normal-weight cohorts are urgently needed.

### 9.3. Need for Larger RCTs with Reproductive Primary Endpoints

Most existing RCTs have examined metabolic outcomes (weight loss, insulin sensitivity, glycemic control) as primary endpoints, with reproductive outcomes (menstrual cyclicity, ovulation, pregnancy) as secondary endpoints. This design limits statistical power to detect differences in reproductive outcomes and may result in selective reporting of positive secondary outcomes. Future trials should:

1. Use reproductive outcomes as primary endpoints: Menstrual cyclicity, ovulation rate, time to pregnancy, clinical pregnancy rate, and live birth rate should be primary endpoints in trials examining GLP-1 RAs for PCOS-related infertility.
2. Enroll adequate sample sizes: Power calculations should be based on reproductive endpoints, with sample sizes adequate to detect clinically meaningful differences in pregnancy and live birth rates.
3. Include longer follow-up: Trials should include at least 6–12 months of follow-up to capture reproductive outcomes and assess durability of metabolic improvements.
4. Include diverse populations: Trials should enroll lean and obese PCOS phenotypes, diverse racial/ethnic groups, and women with different PCOS phenotypes (hyperandrogenic vs. non-hyperandrogenic, ovulatory vs. anovulatory).
5. Include pregnancy registries: Prospective pregnancy registries should be established to systematically collect data on pregnancy outcomes, congenital malformations, and maternal/fetal complications in women exposed to GLP-1 RAs before or during early pregnancy.

### 9.4. Potential for Small-Study Effects

The predominance of small studies in this field raises concerns about small-study effects, where small studies may show larger treatment effects than large studies due to publication bias, methodological limitations, or chance. Formal assessment of small-study effects using funnel plots and statistical tests (e.g., Egger’s test) has not been consistently performed in systematic reviews of GLP-1 RAs for reproductive outcomes. Future systematic reviews should routinely include these assessments to evaluate the potential for publication bias and small-study effects.

## 10. Future Directions and Research Priorities

### 10.1. Mechanistic Validation in Human Tissue

A critical research priority is validation of molecular pathways in human ovarian tissue and clinical samples. Proposed studies include:

1. Human ovarian tissue studies: Examination of GLP-1 receptor expression, localization, and signaling in human ovarian tissue from women with and without PCOS, and assessment of GLP-1 RA effects on steroidogenic enzyme expression, androgen synthesis, and inflammatory markers in human granulosa and theca cells.
2. Clinical biomarker studies: Measurement of circulating and follicular fluid biomarkers (FOXO1, inflammatory cytokines, oxidative stress markers) in women with PCOS before and after GLP-1 RA therapy to assess whether preclinical pathway findings translate to human physiology.
3. Pharmacokinetic studies: Assessment of GLP-1 RA penetration into follicular fluid and ovarian tissue to determine whether therapeutic concentrations are achieved in the ovarian microenvironment.

### 10.2. Clinical Trials in Diverse Populations

Future clinical trials should address current evidence gaps by:

1. Enrolling lean PCOS phenotypes: Dedicated trials in women with BMI < 25 kg/m^2^ to assess whether GLP-1 RAs provide reproductive benefits independent of weight loss.
2. Examining different PCOS phenotypes: Trials stratified by PCOS phenotype (hyperandrogenic vs. non-hyperandrogenic, ovulatory vs. anovulatory, insulin-resistant vs. insulin-sensitive) to identify which phenotypes benefit most from GLP-1 RA therapy.
3. Including diverse racial/ethnic groups: Most existing trials have been conducted in predominantly white or Asian populations; trials in Black, Hispanic, and other underrepresented populations are needed.
4. Examining non-PCOS reproductive disorders: Investigation of GLP-1 RA effects in other obesity-related reproductive disorders (hypothalamic amenorrhea, obesity-related infertility without PCOS).

### 10.3. Pregnancy Safety Studies

Establishing pregnancy safety is a critical priority. Recommended approaches include:

1. Prospective pregnancy registries: Establishment of large, prospective pregnancy registries to systematically collect data on pregnancy outcomes, congenital malformations, and maternal/fetal complications in women exposed to GLP-1 RAs before or during early pregnancy.
2. Pharmacovigilance studies: Enhanced pharmacovigilance and post-marketing surveillance to identify potential safety signals.
3. Animal reproductive toxicology studies: Additional animal studies to characterize dose–response relationships, critical windows of exposure, and mechanisms of embryo-fetal toxicity.

### 10.4. Comparative Effectiveness Research

Head-to-head trials comparing GLP-1 RAs to established PCOS treatments are needed:

1. GLP-1 RAs vs. metformin: Adequately powered RCTs comparing GLP-1 RAs to metformin for reproductive outcomes in women with PCOS.
2. GLP-1 RAs vs. lifestyle modification: Trials comparing GLP-1 RAs to intensive lifestyle modification (diet and exercise) for metabolic and reproductive outcomes.
3. Combination therapy: Trials examining optimal combinations of GLP-1 RAs with metformin, ovulation induction agents, or lifestyle modification.
4. Cost-effectiveness analyses: Economic evaluations to inform clinical decision-making and health policy.

### 10.5. Long-Term Outcomes

Long-term follow-up studies are needed to assess:

1. Durability of reproductive benefits: Whether improvements in menstrual regularity, ovulation, and fertility persist after GLP-1 RA discontinuation or require ongoing therapy.
2. Metabolic outcomes: Long-term effects on insulin resistance, diabetes risk, cardiovascular risk factors, and metabolic syndrome.
3. Offspring outcomes: Long-term health outcomes in children born to women who conceived during or shortly after GLP-1 RA therapy.

### 10.6. Tirzepatide and Multi-Receptor Agonists

Given the promising preclinical data for tirzepatide and the development of triple agonists (GLP-1/GIP/glucagon), research priorities include:

1. Controlled clinical trials of tirzepatide in PCOS: Adequately powered RCTs examining tirzepatide effects on reproductive outcomes in women with PCOS.
2. Mechanistic studies of GIP signaling in ovarian tissue: Investigation of GIP receptor expression and function in human ovarian tissue and assessment of dual GIP/GLP-1 agonism effects on ovarian physiology.
3. Comparative effectiveness of multi-receptor agonists: Head-to-head trials comparing tirzepatide and future triple agonists to selective GLP-1 RAs for metabolic and reproductive outcomes.

## 11. Conclusions

Incretin-based therapies, particularly GLP-1 receptor agonists, represent a biologically plausible and potentially promising approach to improving ovarian function and reproductive outcomes in women with PCOS and obesity-related reproductive dysfunction. The current evidence base suggests that GLP-1 RAs may improve menstrual regularity, hormonal profiles, and spontaneous conception rates, likely mediated primarily through systemic metabolic improvements (weight loss, enhanced insulin sensitivity, reduced inflammation) with potential contributions from direct ovarian effects.

However, the evidence base is characterized by significant limitations, including:—reliance on preclinical mechanistic data without validation in human ovarian tissue; limited high-quality RCTs with small sample sizes and short duration; very high heterogeneity in meta-analyses (I^2^ = 95.6% for menstrual cyclicity); very low to low evidence certainty for several comparisons; restriction to overweight/obese populations with insufficient evidence in lean PCOS; potential publication bias due to small-study effects and lack of formal bias assessment; and insufficient pregnancy safety data.

GLP-1 RAs are not currently approved or guideline-recommended for fertility restoration, and their use in this context remains investigational. Substantial uncertainty remains regarding long-term reproductive safety, optimal patient selection, dosing and duration, comparative effectiveness versus established therapies, and cost-effectiveness.

Future research priorities include: 1. Validation of molecular pathways in human ovarian tissue and clinical samples. 2. Adequately powered, long-term RCTs with reproductive primary endpoints in diverse populations (including lean PCOS phenotypes). 3. Prospective pregnancy registries to establish safety. 4. Head-to-head trials comparing GLP-1 RAs to metformin, lifestyle modification, and ovulation induction agents. 5. Controlled clinical trials of tirzepatide and emerging multi-receptor agonists. 6. Cost-effectiveness analyses to inform clinical decision-making.

Until these evidence gaps are addressed, clinicians should exercise caution when considering GLP-1 RAs for reproductive indications. Use should be limited to research settings or highly selected clinical scenarios (e.g., obese women with PCOS and comorbid type 2 diabetes) with informed consent, comprehensive contraceptive counseling, preconception discontinuation (1–2 months before planned conception), and close monitoring of metabolic and reproductive outcomes.

This review provides a comprehensive, balanced synthesis of current evidence on incretin therapy for ovarian function, clearly distinguishing between established findings and preliminary or hypothesis-generating data, and emphasizing the substantial uncertainties that must be addressed before clinical translation can be recommended.

**Figure 1:**
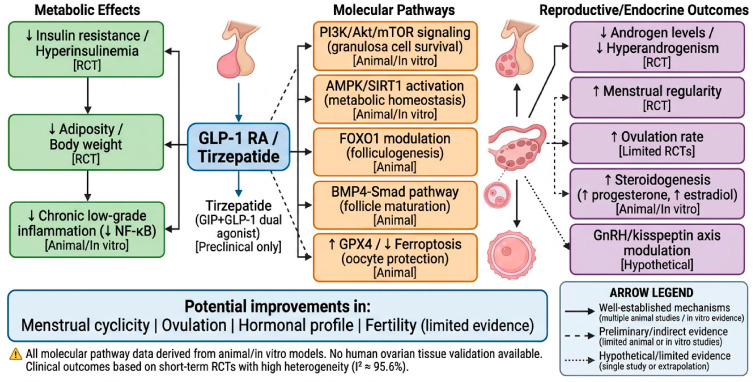
Proposed mechanisms by which incretin-based therapies (GLP-1 receptor agonists and tirzepatide) influence ovarian function in women with PCOS. Solid arrows indicate well-established mechanisms supported by multiple animal studies or in vitro evidence; dashed arrows indicate preliminary or indirect evidence from limited animal or in vitro studies; dotted arrows indicate hypothetical mechanisms based on single studies or extrapolation. Evidence-level tags: [RCT] = randomised controlled trial data available; [Animal/In vitro] = validated only in preclinical models; [Hypothetical] = mechanistic extrapolation without direct experimental validation. ⚠ All molecular pathway data are derived from animal/in vitro models. No human ovarian tissue validation is currently available. Clinical outcomes are based on short-term RCTs with high heterogeneity (I^2^ ≈ 95.6%). AMPK, AMP-activated protein kinase; BMP4, bone morphogenetic protein 4; FOXO1, forkhead box protein O1; GIP, glucose-dependent insulinotropic polypeptide; GLP-1 RA, glucagon-like peptide-1 receptor agonist; GnRH, gonadotropin-releasing hormone; GPX4, glutathione peroxidase 4; mTOR, mechanistic target of rapamycin; NF-κB, nuclear factor kappa-light-chain-enhancer of activated B cells; PCOS, polycystic ovary syndrome; PI3K, phosphoinositide 3-kinase; SIRT1, sirtuin 1. Legend: This schematic illustrates the proposed mechanisms through which GLP-1 receptor agonists may improve ovarian function in women with PCOS. Solid arrows represent pathways supported by human clinical evidence or validated in human tissue. Dashed arrows represent pathways demonstrated in animal models or in vitro systems but not yet validated in human ovarian tissue. Dotted arrows represent hypothetical pathways with limited or indirect evidence. Key pathways include: (1) systemic metabolic improvements (weight loss, improved insulin sensitivity, reduced inflammation) supported by robust human clinical trial evidence; (2) direct ovarian effects via GLP-1 receptors expressed in granulosa and theca cells, supported by receptor expression studies in human ovarian tissue but with mechanistic details derived primarily from animal models; (3) modulation of hypothalamic–pituitary–ovarian axis, with limited direct evidence in humans. The GLP-1 to kisspeptin pathway is depicted with a dotted arrow, reflecting indirect and limited evidence requiring further validation in human studies. Molecular pathways (FOXO1, PI3K/Akt, AMPK, BMP-Smad, GPX4) are shown with dashed arrows, indicating animal/in vitro validation only. Clinicians and researchers should interpret this schematic with awareness of the translational gaps between preclinical and clinical evidence. The arrows indicate stimulatory (↑) and inhibitory (↓) effects of incretin-based therapies on the respective molecular pathways and ovarian functions depicted.

Figure 1 Mechanisms of GLP-1 Receptor Agonist Effects on Ovarian Function.

## Figures and Tables

**Table 1:** Summary of the validation status of molecular pathways through which GLP-1 RAs may influence ovarian function.

Pathway	Evidence Source	Human Ovarian Validation	Key References
FOXO1	DHEA mouse model, in vitro granulosa cells	Not validated	[1]
PI3K/Akt/mTOR	Mouse PCOS model	Not validated	[13]
AMPK/SIRT1/NF-κB	PCOS mouse model	Not validated	[13]
BMP4-Smad	Mouse PCOS model	Not validated	[13]
GPX4/Ferroptosis	Mouse PCOS model	Not validated	[13]
Steroidogenesis	Mixed animal/in vitro	Partial (GLP-1R expression confirmed in human ovary; mechanistic effects not validated)	[1]

## Data Availability

No new data were created or analyzed in this study. Data sharing is not applicable to this article.
